# Subcuticular suture achieves better esthetics, healing, and similar functional outcomes following unicompartmental knee arthroplasty: a single center retrospective comparative study

**DOI:** 10.3389/fmed.2025.1708256

**Published:** 2026-01-06

**Authors:** Changzhi Huang, Canhong Zhang, Shimin Zhang, Nanyi Xu, Lei Zhang, Jiuzao Lin, Xiaoyong Wang

**Affiliations:** 1 Ningde Clinical Medical College of Fujian Medical University, Ningde, Fujian, China; 2 Department of Joint Surgery and Sports Medicine, Ningde Municipal Hospital of Ningde Normal University, Ningde, Fujian, China; 3 Department of Orthopedic Surgery, Quanzhou First Hospital Affiliated to Fujian Medical University, Quanzhou, Fujian, China

**Keywords:** unicompartmental knee arthroplasty, intradermal suture, wound healing, incision suture, cosmetic suture, suture technique

## Abstract

**Aims:**

This study aimed to assess the impact of a modified intradermal suture technique on clinical outcomes and esthetic satisfaction in patients following unicompartmental knee arthroplasty (UKA).

**Methods:**

A total of 80 patients with medial knee osteoarthritis, who underwent UKA between June 2023 and October 2024 at a tertiary academic hospital in Ningde, China, were selected based on inclusion and exclusion criteria. Patients were allocated to either a traditional suture group or a modified suture group, with 40 patients in each group. The cohort consisted of 32 males and 48 females, with a mean age of 67.39 ± 6.33 years (range 57–79) and a mean disease duration of 4.44 ± 1.37 years (range 1–8). Wound healing grade, Hollander Wound Evaluation Score (HWES), and Visual Analog Scale (VAS) were compared 2 weeks post-surgery, while the Patient Scar Assessment Score (PSAS), Observer Scar Assessment Score (OSAS), Range of Motion (ROM), and Hospital for Special Surgery Knee Score (HSS) were assessed 12 weeks post-surgery.

**Results:**

The modified suture group showed a significant reduction in suture time, number of suture reactions, and postoperative hospital stay compared to the traditional group (6.20 vs. 3.65 min, 9 vs. 2, 6.53 vs. 5.38 days; all *p* < 0.05). Although there were fewer postoperative dressing changes, incidences of poor incision alignment, and complications in the modified group, these differences were not statistically significant (*p* > 0.05). Two weeks post-surgery, the modified group exhibited improved HWES scores. At 12 weeks, the modified group demonstrated superior PSAS and OSAS scores (3.38 vs. 4.68, 29.83 vs. 22.40, 23.08 vs. 14.93, all *p* < 0.05). The VAS pain score, ROM, and HSS improved significantly in both groups compared to preoperative values (1.68 vs. 4.33, 116.43 vs. 100.53, 89.23 vs. 52.58 for the modified group, and 1.55 vs. 4.20, 116.38 vs. 101.00, 89.30 vs. 51.65 for the traditional group; all *p* < 0.05). However, no statistically significant differences were found between the groups post-surgery (1.68 vs. 1.55, 116.43 vs. 116.38, 89.23 vs. 89.30, all *p* > 0.05).

**Conclusion:**

The modified intradermal suturing technique, compared to the traditional intermittent method, significantly reduces suturing time and incidence of suture reactions, enhances scar condition, and improves patient satisfaction with the esthetic outcome of the incision. It proves to be an effective suturing technique for UKA patients.

## 1 Introduction

As knowledge of knee osteoarthritis (OA) has advanced, it has been established that early-stage knee OA predominantly affects the medial compartment, with less frequent involvement of the lateral side and patellofemoral joint (1, 2). The anterior medial compartment, also known as anteromedial OA (AMOA), is the most common site of medial OA. AMOA was initially characterized by a distinctive wear pattern, marked by isolated and complete loss of medial knee cartilage, without any damage to the lateral or patellofemoral compartments (3). In the 1970s, unicompartmental knee arthroplasty (UKA) emerged as an alternative to high tibial osteotomy (HTO) for patients with isolated AMOA unresponsive to conservative treatment (4). After nearly 50 years of refinement, UKA has evolved into a highly established surgical technique (5, 6). Recent advances in prosthesis design, surgical techniques, and expanded indications have contributed to the growing global adoption of UKA. Between 2000 and 2009, the number of UKA procedures in the United States increased 6.2-fold, accounting for 4.5% of all knee arthroplasties (7). In the United Kingdom, the proportion of UKA procedures steadily increased from 2014 to 2019, reaching 11.5% in 2019 (8). In Germany, 21,072 UKA procedures were performed in 2018 (9). In China, approximately 23,000 UKA procedures were conducted in 2020, based on incomplete data (10). Among all patients undergoing total knee arthroplasty (TKA), 47% are candidates for partial knee replacement, such as UKA, which is particularly suitable for AMOA (11). A study from India reported a high prevalence (46.94%) of AMOA in patients undergoing primary TKA (12), suggesting they may be potential candidates for UKA. Compared to TKA, UKA offers several advantages, including minimally invasive surgery, fewer complications, bone preservation, shorter hospital stays, faster functional recovery, and higher patient satisfaction (13–17).

As UKA technology becomes more widespread and esthetic outcomes gain importance, both patients and clinicians have higher expectations for the closure of UKA skin incisions (18). The traditional interrupted suture technique often results in a “centipede leg” scar, which significantly impacts esthetics, prompting patients to request cosmetic sutures (19). Additionally, traditional interrupted sutures require removal postoperatively, and clinical experience shows that some patients experience extended hospital stays due to suture removal. Intradermal suturing, a technique initially developed in plastic surgery, has since gained popularity in gynecology, general surgery, and orthopedics (20). With the increasing adoption of the enhanced recovery after surgery (ERAS) protocol, clinicians are more inclined to use intradermal sutures, as they may reduce hospital stays and improve patient satisfaction (21).

The advent of minimally invasive techniques in UKA, coupled with the increasing emphasis on surgical expertise and patient satisfaction, has driven significant advancements in incision suturing technology (5). This development is crucial for optimizing wound healing and minimizing scarring (19). Recent clinical guidelines and studies have further emphasized esthetic outcomes as a key dimension of patient-reported outcome measures (PROMs) in knee arthroplasty (22, 23). A Prospective Single-Blind Randomized Controlled Trial by Masuda et al. (23) demonstrated that skin closure using barbed sutures improved patient satisfaction with wound healing after TKA, and better cosmetic outcomes were associated with better postoperative PROMs. Zhou et al. (24) found that, compared to intermittent suturing, intradermal suturing with barbed suture after TKA offers advantages in terms of safety, efficiency, reduced surgical time, improved scar esthetics, and higher patient satisfaction. The refinement of incision suturing techniques has become a key factor in enhancing postoperative recovery and patient outcomes (25, 26). Inadequate wound healing, leading to knee joint infections, can have severe consequences, potentially determining the success or failure of the surgery (27). Although some studies suggest that topical skin adhesives may reduce wound complications (28, 29), others highlight specific issues, including allergic reactions like allergic contact dermatitis (ACD) (30, 31). This study aims to compare the efficacy of two suturing methods in UKA: traditional intermittent suturing and modified intradermal suturing.

Despite the widespread clinical use of both techniques, the modified intradermal suturing method presented here represents an advancement over conventional approaches. Notably, this innovative technique fully embeds absorbable sutures subcutaneously and achieves uniform distribution of incision tension, thereby eliminating the need for postoperative suture removal and secondary trauma. It addresses issues such as incision dehiscence or suture exposure caused by uneven tension. Clinically, it may reduce suture reactions, shorten the length of hospital stay, and improve esthetics, meeting the demands of UKA patients for function, appearance, and minimally invasiveness. To date, no systematic clinical studies have comprehensively evaluated the use of these methods for UKA incision closure. Therefore, this study aims to assess the impact of modified intradermal suturing on postoperative pain, incision esthetics, patient satisfaction, and knee joint function in UKA. A retrospective analysis was conducted on 80 patients with medial knee OA who underwent UKA at our hospital (a public hospital in the eastern region of Fujian Province, China) between June 2023 and October 2024. Patients’ skin incisions were closed using either modified intradermal sutures or traditional interrupted sutures, and clinical outcomes were compared between the two groups.

## 2 Materials and methods

### 2.1 Patient selection and study cohort

This retrospective study analyzed 80 patients with AMOA who underwent UKA at our hospital between June 2023 and October 2024. As a tertiary comprehensive public hospital affiliated with Ningde Normal University, approximately 100 UKA procedures were performed annually in this institution, representing 25% of all TKA surgeries. Informed consent was obtained from all participants, and the study received approval from the Ethics Committee. The report adheres to the STROBE (Strengthening the Reporting of Observational Studies in Epidemiology) guidelines (32).

Inclusion criteria were as follows: (1) Fulfillment of diagnostic criteria for knee AMOA (3); (2) Correction of knee varus deformity under eversion stress (reversible varus deformity); (3) MRI showing intact anterior and posterior cruciate ligaments, and medial and lateral collateral ligaments; (4) Knee joint range of motion (ROM) > 90°, varus deformity < 15°, and flexion contracture < 15°; (5) Body mass index (BMI) ≤ 40 kg/m^2^ (33); (6) Surgery performed by the same surgical team (with over 10 years of experience), following a standardized procedure; (7) Postoperative rehabilitation conducted by the same group of rehabilitation physicians, with a follow-up period of at least 3 months and complete case data.

**Figure 1:**
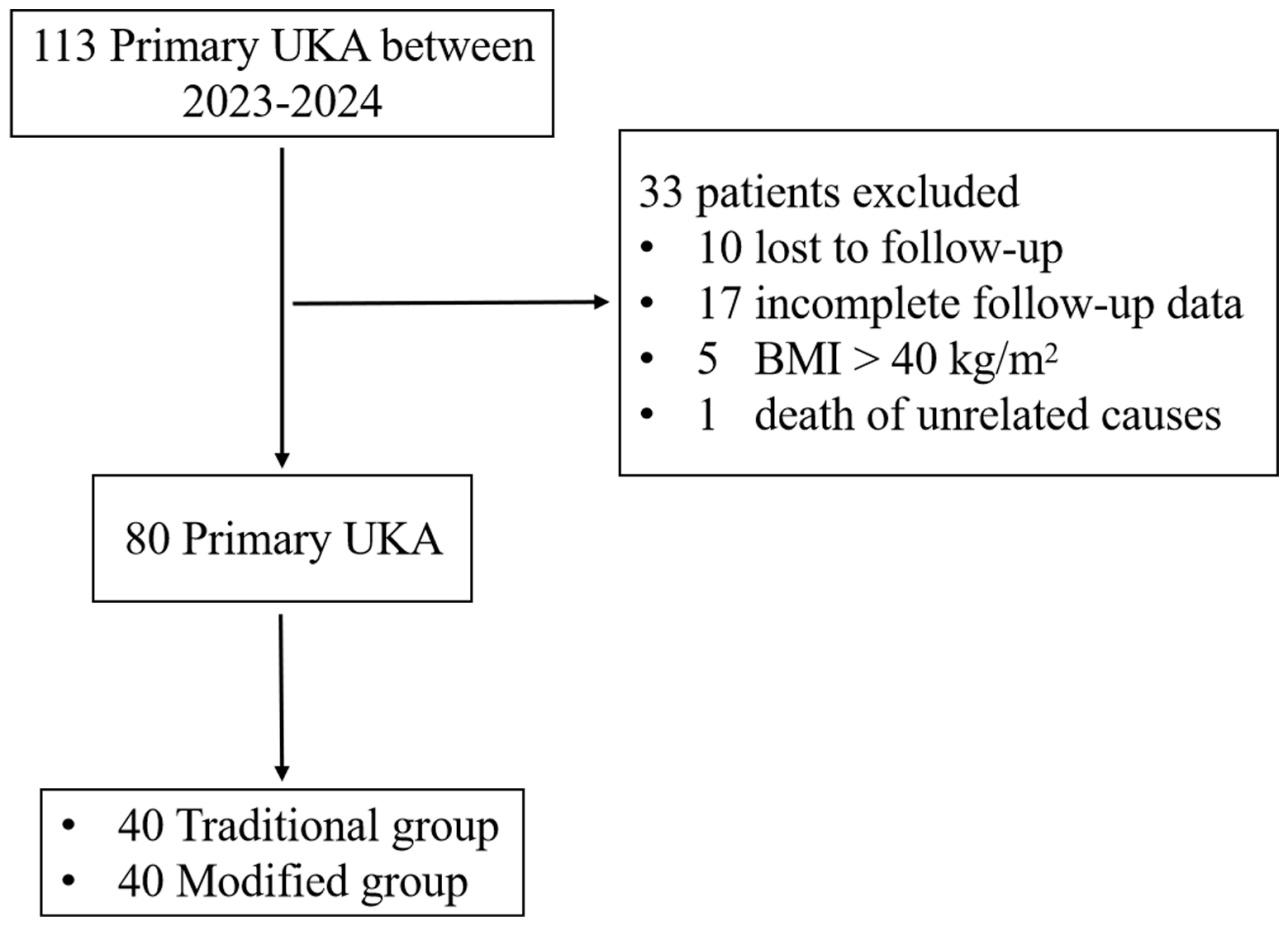
Flowchart of patient selection in the study. UKA, unicompartmental knee arthroplasty; BMI, body mass index.

Exclusion criteria were as follows: (1) Poor skin or soft tissue conditions in the surgical area, such as old scars or sinus tracts; (2) History of severe trauma or previous knee surgery; (3) Severe malnutrition or conditions such as hyperthyroidism, tuberculosis, or tumors; (4) Long-term use of hormones, immunosuppressants, or other medications; (5) American Society of Anesthesiologists (ASA) classification (34): ≥IV. The patient selection process is illustrated in Figure 1.

### 2.2 General information

A total of 80 cases (80 knees) were included in the study, consisting of 32 males and 48 females. The mean age was 67.39 ± 6.33 years (range 57–79 years), and the mean disease duration was 4.44 ± 1.37 years (range 1–8 years). All patients had knee OA classified as grade III or IV according to the Kellgren-Lawrence (K-L) classification (35). Based on the incision closure method, the patients were divided into two groups: the traditional group and the modified group, with 40 cases in each. In the traditional group, the incision was closed using the traditional intermittent suture method, while in the modified group, the modified intradermal suture method was used. No statistically significant differences were found in general characteristics, such as age, gender, BMI, disease duration, OA grade, Charlson comorbidity index (CCI), and ASA grade between the two groups, indicating comparability (*p* > 0.05; Table 1).

**Table 1:** Comparison of baseline characteristics between groups.

Variables	Traditional group (*n* = 40)	Modified group (*n* = 40)	Statistical value	*p-*value
Age (y)	67.48 ± 6.60	67.30 ± 6.13	0.123^a^	0.903
Gender (M/F)	17/23	15/25	0.208^b^	0.648
BMI (kg/m^2^)	28.85 ± 3.60	29.38 ± 3.15	−0.697^a^	0.488
Laterality (Left/Right)	19/21	18/22	0.050^b^	0.823
Disease Course (y)	4.30 ± 1.36	4.58 ± 1.38	−0.898^a^	0.372
K-L Grade (III/IV)	6/34	7/33	0.092^b^	0.762
ASA Grade (I/II/III)	18/20/2	16/20/4	0.794^c^	0.747
CCI	2.55 ± 1.13	3.03 ± 1.21	−1.815^a^	0.073

a Independent-samples t-test. Quantitative data are expressed as mean ± standard deviation.

b Chi-squared test.

c Fisher’s exact test.

BMI, body mass index; K-L, Kellgren-Lawrence; ASA, American Society of Anesthesiologists; CCI, Charlson comorbidity index.

### 2.3 Surgical methods

The patient was positioned supine under either subarachnoid or general anesthesia, with a tourniquet applied to the proximal thigh of the affected side. The knee joint was flexed and placed on a special holder in 40° hip flexion and 30° abduction. The knee was allowed to drop naturally beyond 90° and was then passively flexed to approximately 110°. Routine disinfection and draping procedures were performed. An 8–10 cm medial patellar approach was used. The skin, subcutaneous tissue, and joint capsule were incised in layers to fully expose the surgical field.

Following the standard UKA protocol, tibial and femoral osteotomies were performed sequentially. After prosthesis insertion, knee flexion-extension and lateral stress gap assessments were conducted. The appropriate prosthesis and tibial plateau were then inserted and fixed. After the bone cement set, the wound was irrigated, the tourniquet was released, and hemostasis was achieved. The incision was sutured in layers with the knee at 60° of flexion (36). The joint capsule, muscle, and deep fascia were sutured with #1 coated Vicryl absorbable suture (ETHICON), followed by subcutaneous tissue suturing with 2–0 coated Vicryl absorbable suture (ETHICON).

In the modified intradermal suture group, a 4–0 coated Vicryl absorbable suture with an angled needle (ETHICON) was used for skin closure. The suture penetrated the dermal layer from the subcutaneous layer at one end of the incision, then emerged from the opposite side of the dermis into the subcutaneous layer. A knot was tied and buried in the subcutaneous layer to initiate the suture. A continuous S-shaped suture was then placed within the dermal layer, just beneath the epidermis. Each needle entry point corresponded to the opposite anterior needle exit point, with a slight posterior shift, and the needle distance was approximately 8 mm. Upon reaching the opposite end of the incision, the suture was exited through the skin at the incision’s end and then reinserted through the exit point. The skin was sutured in 8 mm intervals, with three N-shaped sutures used to secure the suture, and the tail of the suture was trimmed close to the skin.

**Figure 2:**
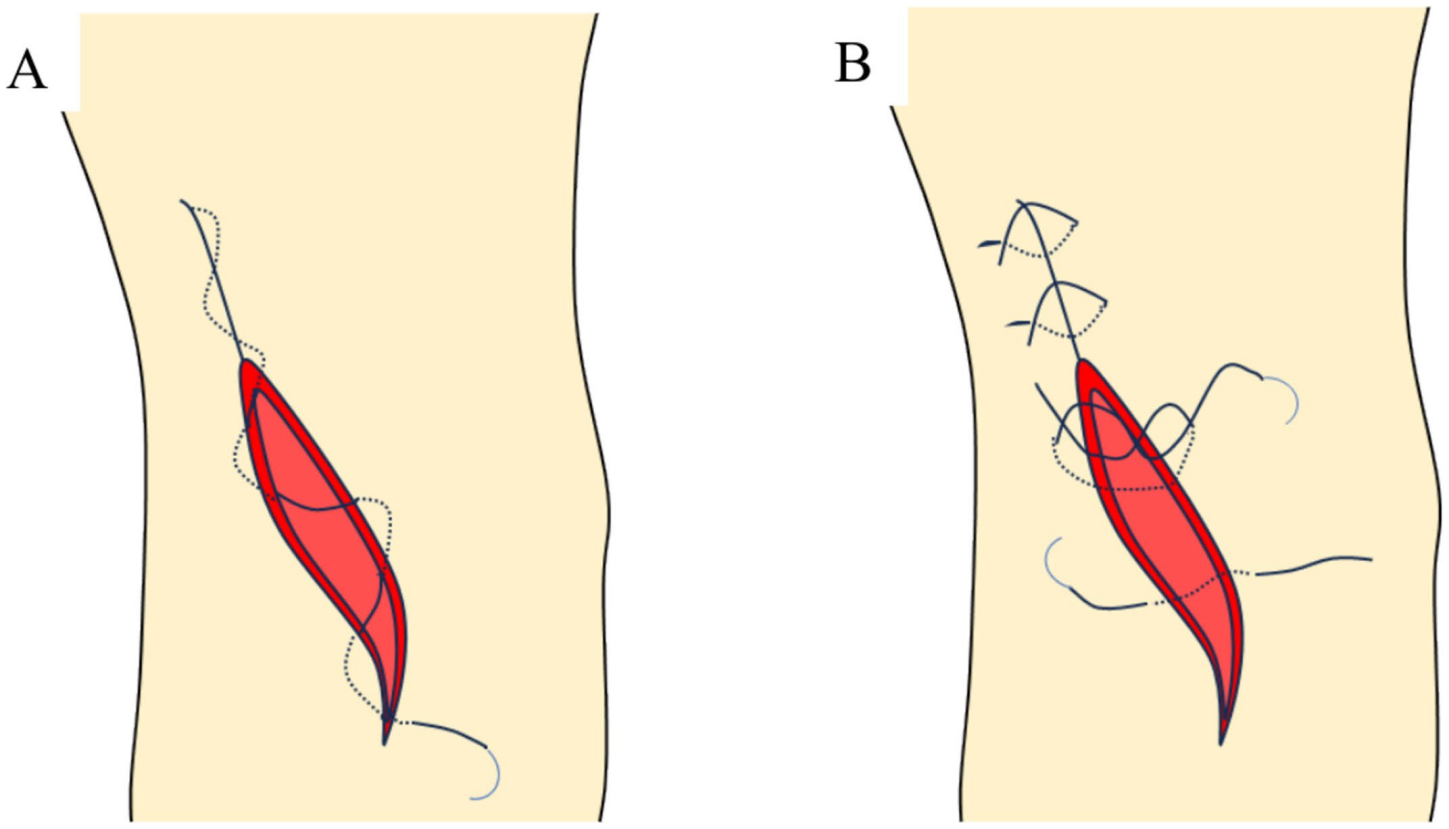
Schematic diagram of incision suture methods. **(A)** Continuous intradermal suture; **(B)** traditional intermittent suture.

**Figure 3:**
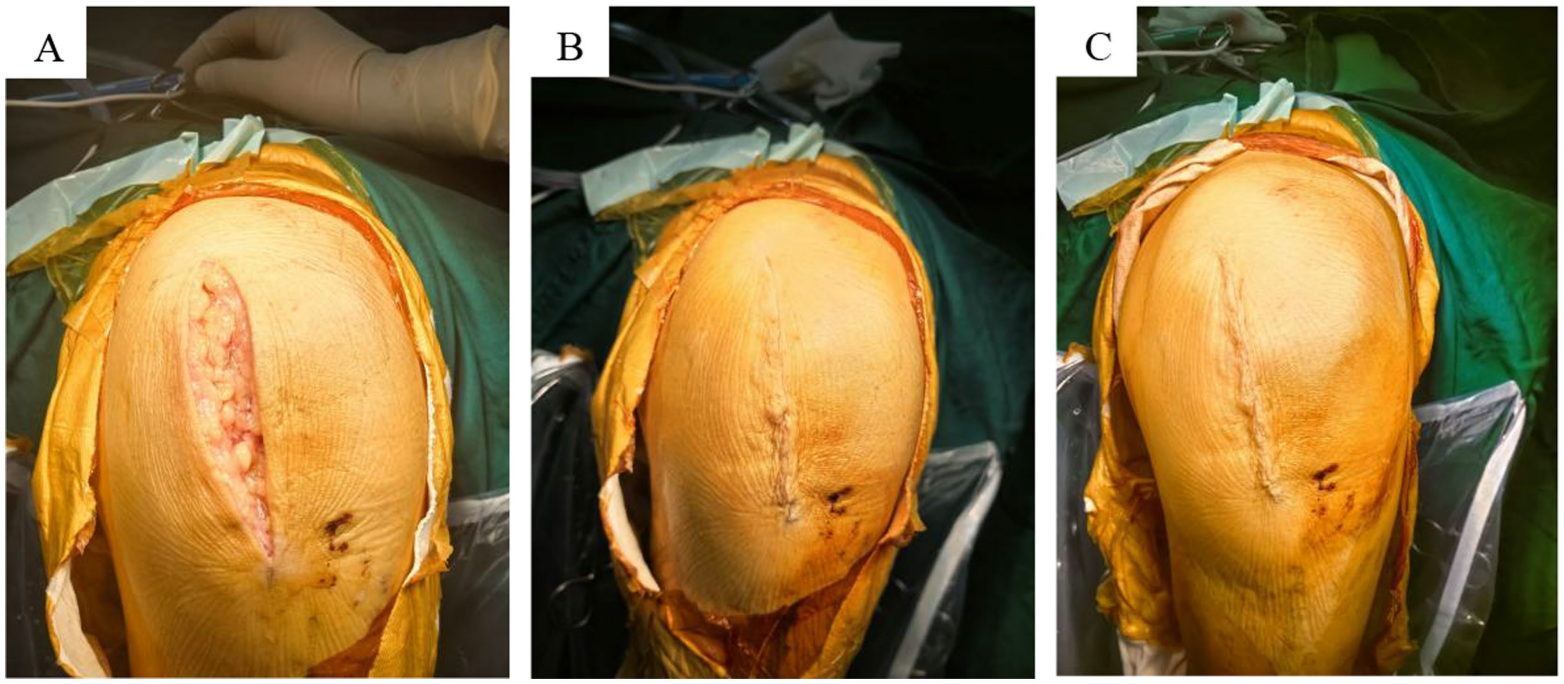
Three layers were sutured. **(A)** Tendon layer closed with absorbable suture; **(B)** subcutaneous layer closed with absorbable suture; **(C)** skin layer closed with absorbable suture.

**Figure 4:**
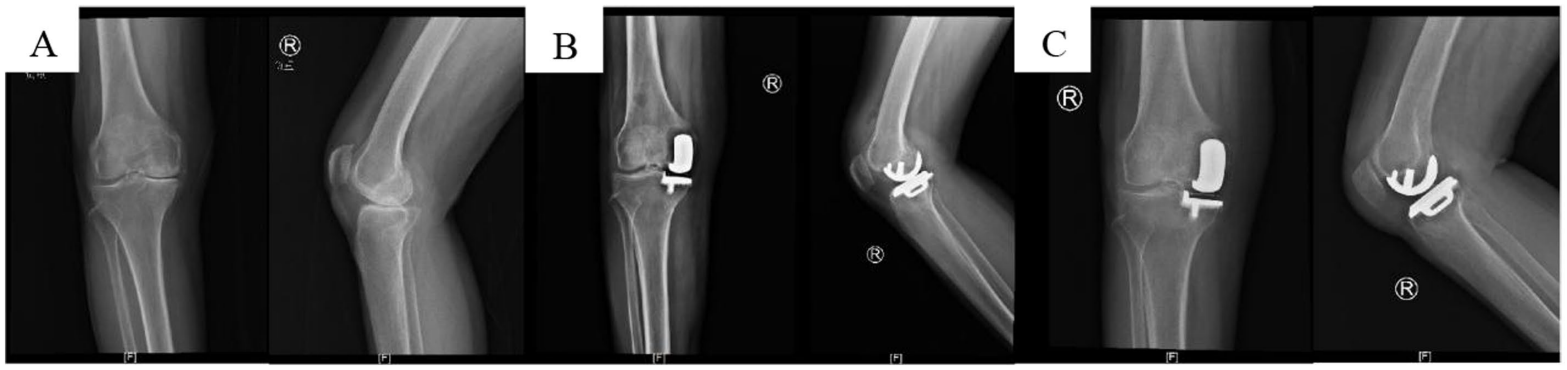
X-ray images of anterior medial knee OA before and after UKA surgery. **(A)** Preoperative image; **(B)** postoperative 2-day image; **(C)** postoperative 3-month image.

In the traditional intermittent suture group, a 4–0 mousse thread was used to intermittently suture the incision. The needle distance was approximately 8 mm, with the distance from the needle eye to the incision about 5 mm. Both groups were operated by the same team of three specialists (two chief physicians and one associate chief physician), each with over 10 years of surgical experience. The implants used were the Biomet Oxford III generation medial unicompartmental knee system (Figures 2–4).

### 2.4 Postoperative management

Postoperatively, Cefazolin was routinely administered within 24 h for infection prevention. To reduce the risk of deep vein thrombosis in the lower limbs, Rivaroxaban was prescribed orally at a dosage of 10 mg once daily for 4 weeks (37). In the operating room, the surgical wound was covered with a hemostatic dressing, and the affected limb was wrapped with cotton pads and compression bandaged with elastic bandages from the ankle to 15 cm proximal to the knee joint. On the first postoperative day, patients began quadriceps contraction and ankle dorsiflexion exercises. The first dressing change was performed on the second postoperative day, with subsequent changes scheduled every 3 to 4 days, depending on the condition of the incision. On the second postoperative day, compression bandages were removed, and patients began knee flexion-extension exercises with the use of walking aids. In the traditional intermittent suture group, sutures were removed between the 12th and 14th postoperative days, based on the healing progress of the incision.

The physical therapy program initiated on the first postoperative day focused on promoting early mobilization. Patients were encouraged to bear or partially bear weight based on their tolerance. The rehabilitation program aimed to help patients regain independence in daily activities through a structured regimen that included bed exercises, ROM exercises, lower limb strengthening, guidance on proper walking technique, and stair climbing practice. Postoperative rehabilitation was supervised by a dedicated team of rehabilitation physicians, each with a minimum of 5 years of clinical experience in rehabilitation.

### 2.5 Follow-up and observation indicators

Two weeks postoperatively, wound healing was assessed using the incision healing criteria (Grade A/B/C), the Hollander wound evaluation score (HWES), and the visual analog scale (VAS) (38, 39). Twelve weeks postoperatively, the incision was evaluated using the Patient Scar Assessment Score (PSAS) (40), Observer Scar Assessment Score (OSAS) (41), ROM, and the Hospital for Special Surgery Knee Score (HSS) (42). Additional data recorded included incision suture time (skin suture only), dressing change frequency, incision complications (e.g., redness, exudation, rupture, subcutaneous hematoma, infection), and postoperative hospital stay for both patient groups.

Incision healing was defined as wound edges approximated without cavitation, showing no separation, and demonstrating well-repaired tissue structure and function (43). Healing was classified into three grades: A, B, and C. Grade A indicated uneventful healing with no adverse reactions. Grade B represented suboptimal healing, characterized by inflammatory responses at the healing site, such as redness, swelling, induration, hematoma, or effusion, but without suppuration. Grade C denoted wound infection with suppuration, requiring debridement or incision drainage (43). Non-grade A healing included both grade B and grade C healing.

The HWES was used to evaluate incision healing, with criteria including incision misalignment, overall esthetic appearance, edge inversion, excessive distortion, and margins exceeding 2 mm in width. Each item was scored 1 point, yielding a total score of 0 to 6, where a score of 6 indicated optimal healing.

The VAS score assessed pain levels, ranging from 0 to 10 points, with 0 representing no pain and 10 indicating severe pain. Scores were classified as follows: < 3 points (mild, tolerable pain), 4–6 points (moderate pain, affecting sleep but still tolerable), and 7–10 points (severe pain, unbearable, affecting sleep and appetite).

The POSAS, which includes the PSAS and OSAS, was used to assess incision scar status (44, 45). The PSAS consists of six items, each scored on a 0–10 scale, while the OSAS includes five items rated on the same scale. Lower scores indicate superior esthetic outcomes.

The HSS score, a widely used tool for evaluating knee joint function, assesses six domains: pain (30 points), function (22 points), ROM (18 points), muscle strength (10 points), knee flexion deformity (10 points), and stability (10 points). The total score ranges from 0 to 100, with higher scores reflecting better knee function.

### 2.6 Statistical analysis

Statistical analysis was performed using SPSS 26.0 software (IBM Corp., Armonk, NY, United States). Quantitative data with a normal distribution are presented as mean ± SD (standard deviation). T-tests were used for inter-group and intra-group comparisons. Count data are presented as the number of cases (n) and percentage (%), with group rate comparisons performed using the Chi-square test or Fisher’s exact test. A *p*-value of less than 0.05 was considered statistically significant.

## 3 Results

### 3.1 Comparison of general indicators

Compared to the traditional group, the modified group showed a significant reduction in suture time, a decrease in suture reactions (such as redness and swelling of the incision due to foreign body reactions of suture material), and a shorter postoperative hospital stay (*p* < 0.05; Table 2). Although the modified group demonstrated fewer postoperative dressing changes, poor incision alignment, incision exudation, and non-grade A healing, these differences were not statistically significant (*p* > 0.05; Table 2).

**Table 2:** Comparison of general perioperative indicators between groups.

Variables	Traditional group (*n* = 40)	Modified group (*n* = 40)	Statistical value	*p*-value
Incision length (mm)	90.68 ± 6.18	91.03 ± 5.39	0.270^a^	0.788
Suture time (min)	6.20 ± 1.20	3.65 ± 0.74	11.441^a^	< 0.001
Dressing changes (n)	2.93 ± 0.62	2.65 ± 0.77	1.765^a^	0.081
Poor incision alignment (n, %)	6 (15)	5 (12.5)	0.105^b^	0.745
Suture reaction (n, %)	9 (22.5)	2 (5)	5.165^b^	0.023
Incision exudation (n, %)	4 (10)	2 (5)	0.396^c^	0.675
Non-grade A healing (n, %)	3 (7.5)	3 (7.5)	1.00^c^	1.000
Postoperative hospitalization time (d)	6.53 ± 1.11	5.38 ± 1.10	4.652^a^	< 0.001

a Independent-samples t-test. Quantitative data are expressed as mean ± standard deviation.

b Chi-squared test.

c Fisher’s exact test.

### 3.2 Comparison of HWES, PSAS, and OSAS scores between groups

**Figure 5:**
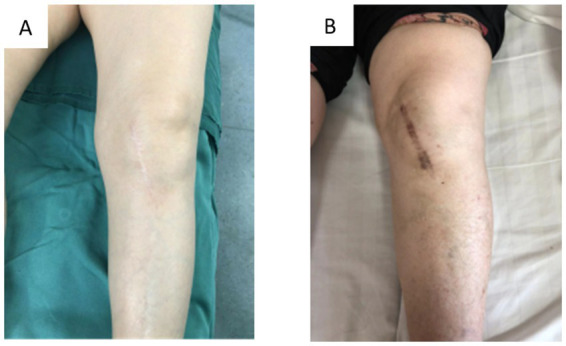
Overview of incision healing. **(A)** Continuous intradermal suture; **(B)** traditional intermittent suture.

Two weeks postoperatively, the HWES score of the modified group was significantly higher than that of the traditional group (*p* < 0.001; Table 3). Additionally, the PSAS and OSAS scores of the modified group were significantly lower than those of the traditional group 12 weeks post-surgery (*p* < 0.001; Table 3). The modified group demonstrated superior wound healing and a more esthetically favorable incision appearance (Figure 5).

**Table 3:** Comparison of HWES, PSAS, and OSAS scores between groups.

Variables	Traditional group (*n* = 40)	Modified group (*n* = 40)	*t*-value	*p-*value
HWES scores	3.38 ± 0.90	4.68 ± 0.57	−7.728	< 0.001
PSAS scores	29.83 ± 1.95	22.40 ± 2.02	16.727	< 0.001
OSAS scores	23.08 ± 2.27	14.93 ± 1.66	18.355	< 0.001

HWES, Hollander wound evaluation score; PSAS, Patient scar self-assessment score; OSAS, Observer scar evaluation score.

At the two-week postoperative mark, the modified group exhibited a significantly higher HWES score compared to the traditional group. By 12 weeks post-surgery, the modified group’s PSAS and OSAS scores were significantly lower than those of the traditional group (*p* < 0.001, Table 3). Statistically significant differences in HWES, PSAS, and OSAS scores between the two groups highlight the benefits of intradermal suturing in improving incision healing esthetics and minimizing scar formation (Figure 5).

### 3.3 Comparison of knee joint pain, ROM, and function between groups before and after surgery

The VAS scores for knee joint pain at 2 weeks post-surgery, as well as the knee joint ROM and HSS function scores at 12 weeks post-surgery, showed significant improvement compared to preoperative levels, with statistically significant differences (*p* < 0.001; Table 4). However, further analysis revealed no significant differences between the two groups in postoperative values for these indicators when compared to their respective preoperative values (*p* > 0.05; Table 4).

**Table 4:** Comparison of knee joint pain, ROM, and functional scores between groups before and after surgery.

Variables	Time point	Traditional group (*n* = 40)	Modified group (*n* = 40)	*t*-value	*p-*value
VAS scores	Preoperative	4.33 ± 0.83	4.20 ± 0.79	0.690	0.492
	Postoperative 2 wk	1.68 ± 0.62	1.55 ± 0.55	0.956	0.342
	*t*-value	15.585	17.667	-	-
	*p-*value	< 0.001	< 0.001	-	-
ROM (°)	Preoperative	100.53 ± 6.23	101.00 ± 7.60	−0.306	0.761
	Postoperative 12 wk	116.43 ± 9.54	116.38 ± 9.20	0.024	0.981
	*t*-value	−8.946	−8.634	-	-
	*p-*value	< 0.001	< 0.001	-	-
HSS scores	Preoperative	52.58 ± 3.80	51.65 ± 4.62	0.979	0.331
	Postoperative 12wk	89.23 ± 3.23	89.30 ± 3.04	−0.107	0.915
	*t*-value	−49.046	−43.754	-	-
	*p-*value	< 0.001	< 0.001	-	-

VAS, Visual analog scale; ROM, Range of motion; HSS, Hospital for Special Surgery Knee Score.

## 4 Discussion

Intradermal suturing techniques are widely used in clinical practice, yielding favorable outcomes. A review of the literature indicates that intradermal suture technology has been extensively applied across various surgical disciplines, including plastic surgery (46), neurosurgery (47), gynecology (48), general surgery (49), and orthopedics (50). It consistently demonstrates beneficial effects such as a lower incidence of incision complications, reduced scar formation, and improved esthetic outcomes.

Absorbable sutures offer significant advantages in intradermal suturing for orthopedic joint surgeries. While both absorbable and non-absorbable sutures are commonly used in clinical practice (51), absorbable sutures eliminate the need for suture removal and reduce patient discomfort, making them increasingly popular (51). In joint surgery, the intradermal suture technique is primarily applied in TKA and total hip arthroplasty (THA), with fewer studies focusing on its use in UKA (38, 52).

The modified intradermal suture combined with absorbable sutures produces superior outcomes in incision healing following UKA. This study aimed to evaluate the impact of the modified intradermal suture technique on clinical outcomes and esthetic satisfaction in UKA patients. The findings substantiate the substantial benefits of the modified intradermal suture, particularly in improving incision healing quality and esthetic results. These results align with current trends in advancing UKA incision suturing techniques both domestically and internationally (52, 53). Vincent et al. (54) demonstrated that interrupted suturing with non-absorbable sutures has several disadvantages, including increased suture knots, a higher risk of infection, prolonged operation time, longer hospital stays, higher costs, and the formation of unsightly “centipede leg” scars. Zhou et al. (24) further confirmed that, compared to intermittent suturing, intradermal suturing with barbed suture after TKA provides advantages in safety, effectiveness, shorter surgical time, improved cosmetic outcomes, and higher patient satisfaction.

The modified intradermal suture technique also offers advantages over other modern wound closure methods commonly used in knee arthroplasty. For instance, topical skin adhesives (e.g., 2-octyl cyanoacrylate) shorten suture time but carry a 5–8% risk of allergic contact dermatitis (30, 31)— a complication absent in our group (0 allergic reactions); barbed sutures demand specialized training and higher costs (54), while our technique uses conventional 4–0 coated Vicryl sutures with a simple S-shaped pattern to lower the learning curve; compared to staple closure, which is linked to higher scar visibility (38), our method achieved superior PSAS and OSAS scores, aligning with patient preferences for “invisible scars.” Its widespread adoption is supported by three core advantages: (1) Technical accessibility—retaining traditional layered suture principles, it only requires brief training on continuous S/N-shaped fixation, with our 10 + year-experience surgical team mastering it after 5–8 cases; (2) Material availability—ETHICON 4–0 coated Vicryl sutures are clinically ubiquitous, with no additional cost versus non-absorbable sutures for traditional closure; (3) ERAS compatibility—reducing suture time and hospital stay, it integrates seamlessly into globally adopted enhanced recovery pathways. Potential adoption barriers (e.g., resistance to changing established suture habits) can be addressed via intraoperative training videos and peer-to-peer mentoring.

The modified intradermal suture, combined with absorbable sutures, promotes incision healing by minimizing scar formation. Regardless of the skin suturing method used, achieving a satisfactory outcome requires proper subcutaneous tissue suturing to ensure anatomical reduction, thereby keeping the skin in a tension-free state during closure (55). While traditional intermittent suturing is effective in providing adequate skin adhesion strength, uneven tension distribution can lead to localized ischemia, increasing the risk of poor wound healing (56). The intradermal suture technique was refined by embedding absorbable sutures continuously within the dermis layer (57). This method offers two key advantages: it allows for tension-free closure of the incision edges and prevents interference with epidermal microcirculation caused by exposed sutures (18, 58). The modified intradermal suture group exhibited a significantly lower incidence of postoperative incision complications (5%) compared to the traditional group (22.5%). This improvement is primarily attributed to the degradation and absorption of the intradermal sutures, which reduced suture reactions and further validated the reliability of this technique. The mechanisms behind the effectiveness of absorbable intradermal sutures in reducing incision scars are multifaceted (59–61). The continuous dermal buried suturing technique fixes incision edges in a tension-free manner, preventing mechanical tension-induced fibroblast abnormalities and collagen disarray (62, 63). This process promotes alignment between the epidermis and dermis, facilitates keratinocyte migration, and inhibits excessive granulation tissue growth (64). The short-term foreign body response accelerates the inflammatory-to-remodeling transition, balancing collagen synthesis (52). Moreover, avoiding suture removal reduces secondary trauma, further lowering the risk of scar hyperplasia (65).

The modified intradermal suture combined with absorbable sutures does not compromise clinical outcomes. The findings of this study showed no statistically significant differences between the two groups in the number of postoperative dressing changes, instances of poor incision alignment, incision exudation, or non-grade A healing. Notably, UKA is regarded as a minimally invasive surgery (66), characterized by a small incision, a short recovery period, and a low overall incidence of complications (22). Further analysis revealed no significant differences between the two groups regarding postoperative knee pain VAS scores, knee joint ROM, or HSS functional scores. This outcome can be attributed to the uniformity of the surgical procedures performed by the same team of physicians and the consistent implementation of postoperative functional exercises by a dedicated rehabilitation therapist. The consistency in postoperative care did not significantly influence efficacy outcomes. Statistically significant differences were observed in suture time, suture reactions, and postoperative hospitalization time between the two groups. These differences were primarily due to the limitations of traditional intermittent suturing, which involves knotting and thread cutting, thereby slowing the suturing process. Additionally, the use of thicker non-absorbable mousse thread for intermittent suturing may have contributed to increased suture reactions at the incision site.

Intermittent sutures require removal and are associated with a heightened suture reaction, often leading to extended observation periods for both patients and physicians, which may result in longer postoperative hospital stays (in some cases, patients are discharged only after suture removal) (67). A statistically significant difference was observed in the HWES, PSAS, and OSAS scores for postoperative incision healing between the two groups, indicating that absorbable thread continuous intradermal suturing is superior to intermittent suturing in terms of incision healing and scar formation. This method not only improves cosmetic outcomes but also enhances patient satisfaction, both from the patient’s self-assessment and the physician’s evaluation.

## 5 Strengths and limitations

This study presents a modified intradermal suture method that offers comparable knee joint function, improved incision esthetics, and smaller scars compared to traditional intermittent suturing, making it a valuable option for clinical practice. The reliability and rationality of the results were supported by the evaluation of multiple indicators.

However, as a retrospective study, potential selection bias and information bias could affect the generalizability of the findings. The limited sample size and short follow-up period inherent in single-center retrospective studies also mean that the long-term efficacy and broader applicability of this method require further validation through prospective multicenter randomized controlled trials. Additionally, the study did not account for confounding factors such as smoking, obesity, and diabetes. Given the high prevalence of these conditions in middle-aged and elderly populations, future studies should incorporate these factors into their design to minimize potential confounding variables, thus enhancing the validity and reliability of the results.

## 6 Conclusion

The modified intradermal suture technique outperforms traditional intermittent suturing in UKA skin closure, offering advantages in surgical efficiency, wound healing, and esthetic outcomes—without compromising knee function over a three-months of follow-up. Given its technical simplicity, material accessibility, and alignment with ERAS and PROMs priorities, this method has strong potential to be integrated into standard UKA surgical protocols, particularly as a routine closure technique for patients with high esthetic expectations. Beyond scar appearance, the reduction in postoperative discomfort and shorter hospital stays further contribute to comprehensive patient satisfaction—addressing the growing emphasis on “functional-esthetic integrated recovery” in modern orthopedics. Future prospective multicenter studies with longer follow-up will help validate its long-term efficacy and solidify its role in standard practice.

## Data Availability

The original contributions presented in the study are included in the article/supplementary material, further inquiries can be directed to the corresponding authors.
